# Optimization of the Aqueous Extraction of Phenolic Compounds from Olive Leaves

**DOI:** 10.3390/antiox3040700

**Published:** 2014-10-23

**Authors:** Chloe D. Goldsmith, Quan V. Vuong, Costas E. Stathopoulos, Paul D. Roach, Christopher J. Scarlett

**Affiliations:** 1 School of Environmental & Life Sciences, University of Newcastle, Ourimbah, NSW 2258, Australia; E-Mails: vanquan.vuong@newcastle.edu.au (Q.V.V.); paul.roach@newcastle.edu.au (P.D.R.); c.scarlett@newcastle.edu.au (C.J.S.); 2 Faculty of Bioscience Engineering, Ghent University Global Campus, Incheon 406-840, South Korea; E-Mail: costas.stathopoulos@ghent.ac.kr

**Keywords:** olive leaves, phenolic compounds, green extraction solvents, waste valorisation, *Olea europaea*, response surface methodology (RSM)

## Abstract

Olive leaves are an agricultural waste of the olive-oil industry representing up to 10% of the dry weight arriving at olive mills. Disposal of this waste adds additional expense to farmers. Olive leaves have been shown to have a high concentration of phenolic compounds. In an attempt to utilize this waste product for phenolic compounds, we optimized their extraction using water—a “green” extraction solvent that has not yet been investigated for this purpose. Experiments were carried out according to a Box Behnken design, and the best possible combination of temperature, extraction time and sample-to-solvent ratio for the extraction of phenolic compounds with a high antioxidant activity was obtained using RSM; the optimal conditions for the highest yield of phenolic compounds was 90 °C for 70 min at a sample-to-solvent ratio of 1:100 g/mL; however, at 1:60 g/mL, we retained 80% of the total phenolic compounds and maximized antioxidant capacity. Therefore the sample-to-solvent ratio of 1:60 was chosen as optimal and used for further validation. The validation test fell inside the confidence range indicated by the RSM output; hence, the statistical model was trusted. The proposed method is inexpensive, easily up-scaled to industry and shows potential as an additional source of income for olive growers.

## 1. Introduction

Adherence to a Mediterranean-style diet has been associated with a reduced risk for cardiovascular disease and certain types of cancers [1]. These associations have been linked, in part, to the high consumption of olive oil, more specifically, the consumption of the unique phenolic compounds found in olive oil [2,3,4]. The same compounds believed to be responsible for the health-promoting properties attributed to olive oil consumption have also been identified in olive leaves [5]. Hence, the potential applications for the health promoting compounds extracted from olive leaves are extensive. These include their use as food additives or health supplements, as well as their continued use in future research into potential anti-cancer [6], anti-inflammatory [7] or anti-fungal [8] agents. It is therefore important to optimize the extraction of these compounds. An understanding of the parameters affecting the extraction of phenolic compounds is paramount to establishing the foundations for this future work.

Mediterranean countries account for around 98% of the world’s olive cultivation (approximately ten million hectares); they produce about 1.9 million metric tonnes per annum of olive oil and 1.1 million tonnes of table olives [9]. Olive leaves are an agricultural waste of the olive oil and table olive production industry. This waste is produced as a result of pruning olive trees during the growing season, as well as accounting for approximately 10% of the weight of materials received by olive mills. Currently, this by-product is not profitable, given that in many countries, olive leaves are used as animal feed or simply burned with excess branches gathered from pruning [10,11]. Many olive oil producers even charge a fee to the olive farmer for the disposal of olive leaves.

The market for natural additives and ingredients is rapidly growing, with some natural products obtaining high prices. Moreover, the possible toxicity of certain synthetic compounds [5,12] has led to an increased interest in natural product research from the cosmetic, pharmaceutical and food additive industries. This has led to improved extraction, fractionation and purification technologies being developed in the last few years. However, these modern purification and separation technologies can be expensive and sometimes hazardous, rendering it near impossible for farmers to profit directly.

A number of methods have been proposed for the extraction of phenolic compounds from olive leaves, including the use of advanced technologies, such as microwave, pressurized liquid extraction and ultra-sonic extraction methods [13,14,15]. However, these practices can often have high energy costs and lead to the production of excessive solvent waste, which can be more hazardous to dispose of than the actual agricultural waste itself. Therefore, there is a need for the development of “green” extraction procedures. Water is a cheap, non-hazardous polar extraction solvent. It has been shown to efficiently extract a vast array of phenolic compounds with high antioxidant activities from a number of plant materials [16,17,18].

Therefore, in the present study, we aimed to optimize the extraction of phenolic compounds from olive leaves using the inexpensive, non-hazardous and easily obtainable solvent, water. The parameters of time, temperature and sample-to-solvent ratio were chosen for optimization, as they are easy for farmers or processors to control. The influence of these extraction parameters on antioxidant activity was also investigated.

## 2. Experimental Section

### 2.1. Materials and Reagents

Folin–Ciocalteu’s reagent, sodium carbonate, gallic acid, 1,1-diphenyl-2-picrylhydrazyl (DPPH), 6-hydroxy-2,5,7,8-tetramethylchroman-2-carboxylic acid (trolox), 2,4,6-Tris(2-pyridyl)-*s*-triazine (TPTZ), ferric chloride, sodium acetate, acetic acid, copper (II) chloride, ammonium acetate (NH_4_Ac), neocuproine methanol and ethanol were purchased from Sigma Aldrich (Castle Hill, Australia). Ultra-pure (type 1) de-ionized (DI) water was prepared by reverse osmosis and filtration using a Mili-Q direct 16 system (Milipore Australia Pty Ltd., North Ryde, Australia).

### 2.2. Sample Preparation

Corregiola olive leaves were obtained from Houndsfield Estate in the Hunter Valley of NSW Australia. The leaves were dried at 120 °C for 90 min according to [19], ground to a size of 0.1 mm and stored at −20 °C until further analysis.

### 2.3. Response Surface Methodology (RSM)

The RSM with the Box–Behnken design was then employed to design the experiment to investigate the influence of three independent parameters, temperature, time and sample-to-solvent ratio, on the extraction of total phenolic compounds (TPC) and on the antioxidant activity of the resultant extracts. The optimal ranges of temperature (70–90 °C), time (50–70 min) and sample-to-solvent ratio (1:10–1:100 g/mL) were determined based on preliminary experiments. The independent variables and their code variable levels are shown in Table 1. To express the TPC or antioxidant capacity as a function of the independent variables, a second-order polynomial equation was used as follows and as previously described by Vuong *et al.* [20]: $Y=\beta_{0}+\sum_{i=1}^{k}\beta_{i}X_{i}+\sum_{i=1}^{k=1}\sum_{j}^{k}\beta_{ij}X_{i}X_{j}+\;\sum_{i=1}^{k}\beta_{ii}X_{i}^{2}$, where various *X_i_* values are independent variables affecting the response *Y*; *β*_0_, *β_i_*, *β_ii_*, and *β_ij_* are the regression coefficients for the intercept and the linear, quadratic and interaction terms, respectively, and *k* is the number of variables.

### 2.4. Total Phenolic Compounds

The TPC was determined according to Thaipong *et al.* [21]. Briefly, the appropriately diluted samples (300 μL) were added to Folin–Ciocalteu’s reagent (300 μL) and left to equilibrate for 2 min before adding 2.4 mL of 5% sodium carbonate solution and incubating in the dark for 1 h. Absorbance was then read at 760 nm using a UV spectrophotometer (Varian, Melbourne, Australia). Gallic acid was used as the standard, and results were expressed as mg of gallic acid equivalents per g of sample (mg GAE/g).

**Table 1:** Values of the independent parameters and their coded forms with their symbols employed in RSM for optimization of olive leaf extraction using water.

Independent Parameters	Symbols of the Parameters	Original Values of the Parameters	Parameter Coded Forms *
**Temperature (°C)**	*X*_1_	70	−
		80	0
		90	+
**Time (min)**	*X*_2_	50	−
		60	0
		70	+
**Ratio (mg/mL)**	*X*_3_	10	−
		55	0
		100	+

* Parameter coded forms −, 0 and + are the minimum point, centre point and maximum point (respectively) for the independent parameters temperature, time and ratio.

### 2.5. Antioxidant Activity Assays

Three assays were employed in order to assess the antioxidant activity of the olive leaf extracts:

For the ferric reducing antioxidant power (FRAP) assay, the extract was diluted within the appropriate range, and then, their ferric ion reducing capacity was determined according to Thaipong *et al.* [21].

Stock solutions were: (1) 300 mM acetate buffer; (2) 10 mM TPTZ solution in 40 mM HCL; (3) 20 mM FeCl_3_ solution. The fresh working solution was prepared by mixing 25 mL acetate buffer, 2.5 mL TPTZ solution and 2.5 mL FeCl_3_ and then warming to 37 °C. Olive leaf extracts, standards and blanks (150 µL) were then added to 2.85 mL of the working FRAP solution and left to incubate in the dark at 37 °C for 30 min. Absorbance was read at 593 nm. Results were expressed as mg trolox equivalents per gram of sample dry weight (mg Trolox Equivalents (TE)/g).

For the cupric reducing antioxidant capacity (CUPRAC) assay, the extracts were diluted within the appropriate range, and their cupric ion reducing capacity was determined as described by Apak *et al.* [22].

The stock solutions were: (1) 10 mM CuCl_2_ solution; (2) ammonium acetate buffer at pH 7.0; (3) 7.5 mM neocuproine (Nc) solution in 95% ethanol. A working solution of the three reagents (1:1:1 v/v) was prepared, 3 mL of which was added to 1.1 mL of the diluted extracts, standards and blanks and left to react in the dark for 1 hour. Absorbance was read at 450 nm. Results were expressed as mg of trolox equivalents per gram of sample dry weight (mg TE/g).

The DPPH free radical scavenging activity of the extracts was analysed using the 1,1-diphenyl-2-picrylhydrazyl (DPPH) assay, as described by Vuong *et al.* [23]. Briefly, the appropriately diluted samples, standards and blank (150 µL) were added to 2.85 mL of DPPH working solution (made to an absorbance of 1.1 ± 0.01 at 760 nm) and left to react in a dark at room temperature for 3 h. Trolox was used as a standard. The results were expressed as mg of trolox equivalents per g of sample dry weight (mg TE/g).

### 2.6. Statistical Analysis

The RSM experimental design and analysis was conducted using JMP software (Version 11, SAS, Cary, NC, USA). The software was also used to establish the model equation, graph the 3D plot with 2D contour of the responses and to predict the optimum values for the three response variables in order to obtain the maximum TPC level. All experiments were carried out in triplicate.

## 3. Results and Discussion

### 3.1. Fitting the Models for the Prediction of Total Phenolic Compounds and Antioxidant Capacity

The experimental design is presented in Table 1, while Table 2 indicates the effects of temperature, time and the ratio of sample-to-solvent on the extraction of TPC from olive leaves using water. The predicted yield of TPC ranged from 22.36 to 38.25 mg GAE/g depending on the combination of extraction parameters.

**Table 2:** Analysis of variance for the determination of the fit of the model. TPC, total phenolic compounds; FRAP, ferric reducing antioxidant power; CUPRAC, cupric reducing antioxidant capacity; PRESS, predicted residual sum of squares.

Sources of Variation	TPC	Antioxidant Capacity		
		FRAP	CUPRAC	DPPH
Lack of fit (*p*-value)	0.1991	0.0168 *	0.1369	0.1377
*R*^2^	0.8	0.95	0.97	0.92
Adjusted *R*^2^	0.44	0.87	0.92	0.78
PRESS	1149.1	1500.72	1097.5	1988.1
F-ratio of model	2.2025	11.54	19.6	6.639
*p* of model > F	0.1991	0.0075 *	0.0022 *	0.0258 *

* Significant difference with *p* < 0.05.

**Figure 1:**
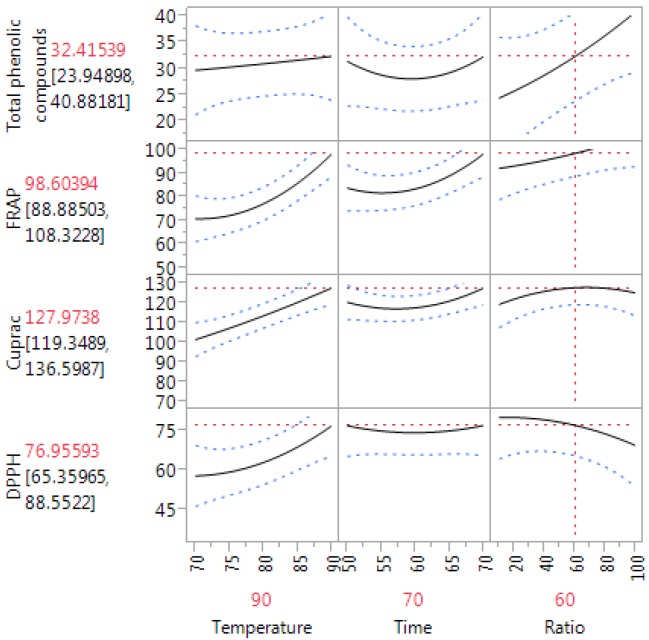
Prediction profiler plots for the effects of the test parameters on the extraction of phenolic compounds from olive leaves.

Table 2 shows the reliability of the RSM mathematical model in predicting optimal variances and accurately representing the real interrelationships between the selected parameters. The results for the analysis of variances of the Box–Behnken design are shown in Table 2. Figure 1 indicates the correlation between the predicted and experimental values.

Figure 1 and Table 2 indicate that there was no significant difference between the actual and predicted values for TPC (*p* > 0.05). Furthermore, the coefficient of determination (*R*^2^) value for the correlation between the predicted and actual values was 0.8, indicating that the model can predict 80% of the actual data for TPC. Table 2 also showed that the “lack of fit” for the model was also not significant (*p* = 0.1991). In addition, the PRESS (predicted residual sum of squares) was 1149.1 and the F-ratio was 2.2025. PRESS is a measure of how well each point fits into the experimental design, further identifying the appropriateness of the model’s fit.

It was therefore concluded that the second-order polynomial equation for the following three independent variables could be used: temperature (*X*_1_), time (*X*_2_) and sample-to-solvent ratio (*X*_3_). The predictive equation for the response of total phenolic compounds (*Y*) was as follows:

*Y* = 26.02 + 1.31 *X*_1_ + 0.42 *X*_2_ + 4.88 *X*_3_ − 0.14 *X*_1_* X*_2_ + 1.42 *X*_1_* X*_3_ + 1.91 *X*_2_* X*_3_ + (0.09 *X*_1_)^2^ + (3.79 *X*_2_)^2^ + (1.23 *X*_3_)^2^ (1)

**Figure 2:**
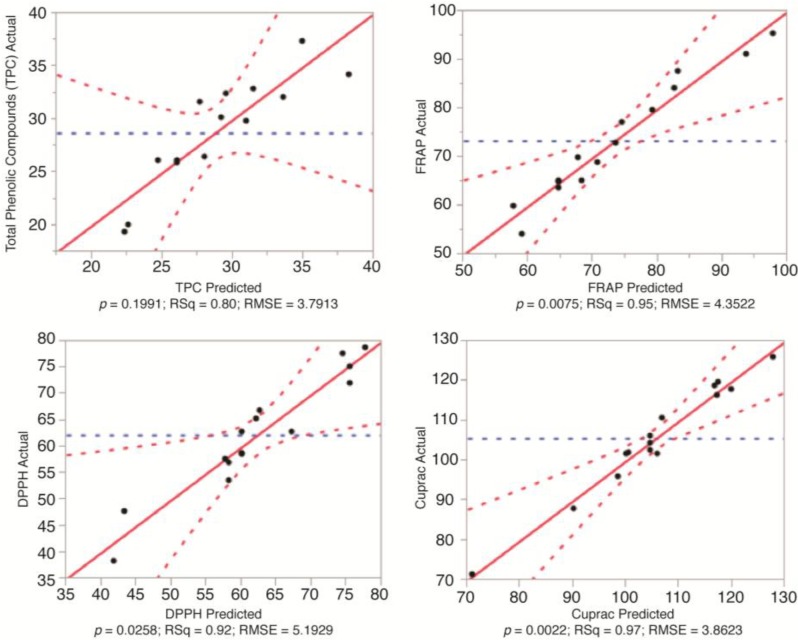
Correlation between the actual and the predicted values for the total phenolic compounds (TPC) and antioxidant capacity of olive leaf water extract (FRAP, DPPH and CUPRAC).

**Figure 3:**
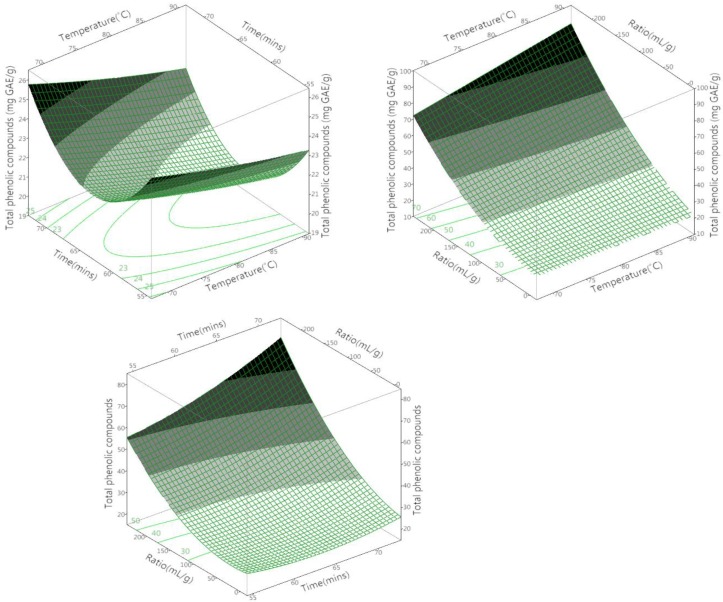
3D response surface and 2D contour plots for the effects of the test parameters on total phenolic compounds.

**Figure 4:**
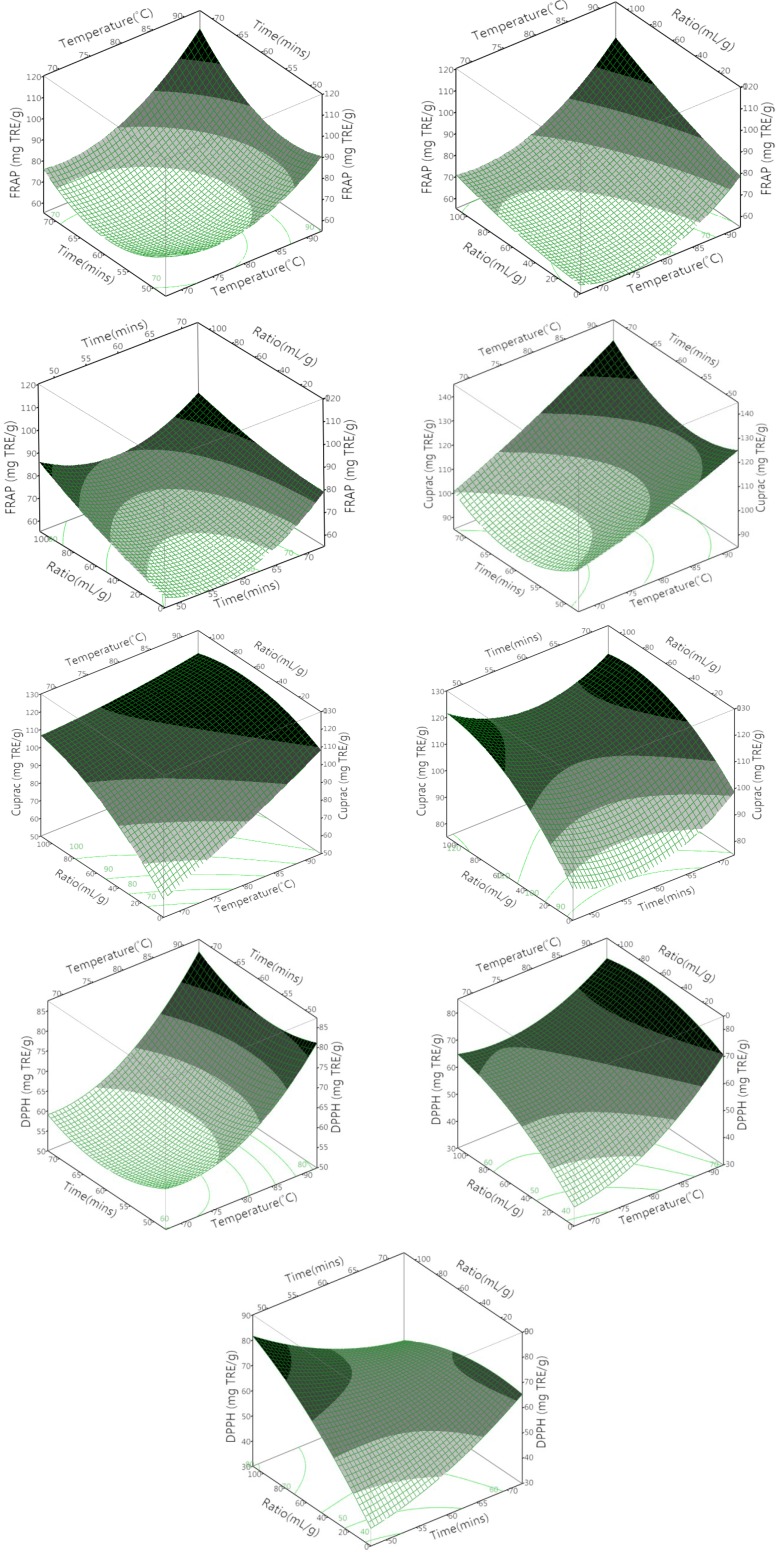
3D response surface and 2D contour plots for the effects of the test parameters on antioxidant activity.

The model fit for the antioxidant activity of the olive leaf extract was also investigated. Figure 2, Figure 3 and Figure 4 show the relationship between the actual and predicted values, while Table 2 represents the analysis of variance results for the determination of the fit of the model. The *p*-values for the model fit were 0.0168, 0.1369 and 0.1377 for FRAP, CUPRAC and DPPH, respectively. This shows that there was no difference between actual and predicted values for CUPRAC and DPPH. However, there was a significant difference between the actual and predicted values for FRAP.

The coefficients of determination were 0.95, 0.97 and 0.92 for FRAP, CUPRAC and DPPH, respectively. This highlighted the close correlation between the actual and predicted values. This relationship is further supported with the values for PRESS and the F-ratios of the model: 1500.72 and 11.54 for FRAP, 1097.5 and 19.6 for CUPRAC and 1988.1 and 6.639 for DPPH, respectively. This indicated that the mathematical models were reliable predictors of the antioxidant activity of the olive leaf water extracts. Therefore, the following second order polynomials could be used:

FRAP:

*Y* = 64.66+ 10.51 *X*_1_ + 4.58 *X*_2_ + 7.45 *X*_3_ + 3.05 *X*_1_*X*_2_ + 2.16 *X*_1_*X*_3_ − 2.66 *X*_2_*X*_3_ + (7.39 *X*_1_)^2^ + (7.64 *X*_2_)^2^ + (1.4 *X*_3_)^2^ (2)

CUPRAC:

*Y* = 104.53+ 11.76 *X*_1_ + 1.91 *X*_2_ + 11.31 *X*_3_ + 2.06 *X*_1_* X*_2_ − 6.14 *X*_1_* X*_3_ − 2.27 *X*_2_* X*_3_ + (1.01 *X*_1_)^2^ + (6.45 *X*_2_)^2^ − (5.33 *X*_3_)^2^ (3)

DPPH:

*Y* = 60.08+ 9.29 *X*_1_ + 0.39 *X*_2_ + 7.02 *X*_3_ + 0.68 *X*_1_* X*_2_ − 3.4 *X*_1_* X*_3_ − 8.98 *X*_2_* X*_3_ + (4.43 *X*_1_)^2^ + (2.71 *X*_2_)^2^ − (3.03 *X*_3_)^2^ (4)

### 3.2. The Effect of the Different Variables on the Total Phenolic Compounds

Table 3 presents the linear regression coefficients and indicates their statistical significance. Temperature, time and ratio were all shown to have a positive influence on the extraction of TPC. However, the only parameter to significantly affect the extraction efficiency was the sample-to-solvent ratio (*p* = 0.01). Temperature and time had no significant effect on TPC (*p* > 0.05), nor did any of the various combinations of factors (temperature × time, temperature × ratio or time × ratio) (Table 3). This was unexpected, since time has previously been shown to have a significant effect on the extraction of TPC from olive leaves when using ultrasonic assistance [15]. Extraction time has also been identified as a significant extraction parameter for the extraction of natural polyphenols from wheat bran [24]. However, in both of these studies, the use of advanced technologies could account for the observed differences.

**Table 3:** The analysis of variance for the experimental results.

Parameter	DF	TPC		Antioxidant Capacity					
				Frap		DPPH		CUPRAC	
		F	Prob > F	F	Prob > F	F	Prob > F	F	Prob > F
***β*_0_**	1	26.02	<0.0001	64.66	<0.0001	60.08	<0.0001	104.53	<0.0001
***β*_1_**	1	1.31	0.37	10.51	0.001 *	9.29	0.004 *	11.76	0.0003 *
***β*_2_**	1	0.42	0.77	4.58	0.031 *	0.39	0.84	1.91	0.22
***β*_3_**	1	4.88	0.01 *	7.45	0.005 *	7.02	0.01 *	11.31	0.0004 *
***β*_12_**	1	−0.14	0.94	3.05	0.22	0.68	0.8	2.06	0.34
***β*_13_**	1	1.42	0.49	2.16	0.37	−3.4	0.25	−6.14	0.02 *
***β*_23_**	1	1.91	0.36	−2.66	0.28	−8.98	0.02 *	−2.27	0.29
***β*_11_**	1	0.09	0.96	7.39	0.02 *	4.43	0.16	1.01	0.63
***β*_22_**	1	3.79	0.11	7.64	0.02 *	2.71	0.36	6.45	0.02 *
***β*_33_**	1	1.23	0.56	1.4	0.56	−3.03	0.31	−5.33	<0.05 *

* Significantly difference with *p* < 0.05; *β*_0_: intercept; *β*_1_, *β*_2_ and *β*_3_: linear regression coefficients for temperature, time and ratio; *β*_12_, *β*_13_ and *β*_23_: regression coefficients for interaction between temperature × time, temperature × ratio and time × ratio; *β*_11_, *β*_22_ and *β*_33_: quadratic regression coefficients for temperature × temperature, time × time and ratio × ratio; Prob = probability.

The sample-to-solvent ratio was shown to have a significant effect on the extraction of TPC. This is consistent with mass transfer principles, which outline that the concentration gradient (the driving force) is higher when there is more solvent present, leading to higher diffusion rates.

### 3.3. The Effect of the Different Variables on Antioxidant Activity

The temperature and ratio were both found to significantly impact the antioxidant activity of the olive leaf extract measured via FRAP, CUPRAC and DPPH (*p* = 0.001, 0.004, 0.0003, respectively). However, time was only shown to significantly affect the antioxidant capacity measured via FRAP. The temperature × ratio had a negative influence on the DPPH measurements (*p* < 0.05).

### 3.4. Optimization of Aqueous Extraction Conditions for Maximizing the Total Phenolic Content and Antioxidant Capacity of Olive Leaf Extract

Based on the predictive models shown in Figure 3 and Figure 4 the optimal conditions for the aqueous extraction of phenolic compounds were a temperature of 90 °C for 70 min at a sample-to-solvent ratio of 1:100 g/mL. These conditions were the same for the optimization of antioxidant capacity via FRAP. However, the optimal conditions for CUPRAC and DPPH varied slightly (CUPRAC: temperature 90 °C, time 70 min, sample-to-solvent ratio of 1:60 g/mL, DPPH: temperature 90 °C, time 70 min, sample-to-solvent ratio of 1:20 g/mL). Therefore, the extraction conditions of a temperature at 90 °C for 70 min and at a sample-to-solvent ratio of 1:60 g/mL were chosen for the extraction of phenolic compounds, as the extracts also displayed a high level of antioxidant activity. Furthermore, consuming less extraction solvent is practical from an economic point of view. For this reason, the sample-to-solvent ratio of 1:60 g/mL was used for validation. Increases in antioxidant activity with increasing temperature have previously been linked to the thermal degradation of higher molecular weight compounds into lower molecular weight ones [25,26]. This is one example of the non-specificity of the Folin–Ciocalteu method.

In order to validate the conditions predicted by the models, these extraction conditions (temperature 90 °C, time 70 min, sample-to-solvent ratio of 1:60 g/mL) were tested. The resulting values fell inside of the predicted ranges for TPC and all three antioxidant capacity assays (Table 4). These conditions are therefore proposed as optimal for the aqueous extraction of phenolic compounds with a high antioxidant capacity from olive leaves.

**Table 4:** Validation of the experimental model. GAE, gallic acid equivalents.

Assay	Values of TPC and Antioxidant Capacity	
	Predicted	Experimental (*n* = 3)
TPC (mg GAE/g)	32.42 ± 8.66	32.4 ± 2.06
FRAP (mg TE/g)	98.6 ± 9.71	91.03 ± 6.13
DPPH (mg TE/g)	76.96 ± 11.56	85.26 ± 3.54
CUPRAC (mg TE/g)	127.97 ± 8.62	121.97 ± 5.45

## 4. Conclusions

The optimal conditions for the aqueous extraction of phenolic compounds from olive leaves were proposed to be at 90 °C for 70 min at a sample-to-solvent ratio of 1:60 g/mL. Using olive leaves as a starting material for the extraction of phenolic compounds via this simple and inexpensive method constitutes a viable use for this agricultural waste product and may potentially serve as an additional source of income for olive growers/olive oil producers.
